# Is A Healthy Diet Associated with Lower Anthropometric and Glycemic Alterations in Predisposed Children Born from Mothers with Gestational Diabetes Mellitus?

**DOI:** 10.3390/nu11030570

**Published:** 2019-03-07

**Authors:** Camille Dugas, Mélissa Bélanger, Julie Perron, S. John Weisnagel, André Tchernof, Isabelle Marc, Julie Robitaille

**Affiliations:** 1 School of Nutrition, Laval University, 2425 rue de l‘Agriculture, Quebec City, QC G1V 0A6, Canada; Camille.dugas.1@ulaval.ca (C.D.); melissa.belanger.5@ulaval.ca (M.B.); andre.tchernof@crcriucpq.ulaval.ca (A.T.); 2 Institute of Nutrition and Functional Foods (INAF), Laval University, 2440 Boulevard Hochelaga, Quebec City, QC G1V 0A6, Canada; julie.perron@fsaa.ulaval.ca; 3 Endocrinology and Nephrology Axis, CHU de Québec Research Center, 2705 Boulevard Laurier, Quebec City, QC G1V 4G2, Canada; john.weisnagel@crchul.ulaval.ca (S.J.W.); isabelle.marc@crchudequebec.ulaval.ca (I.M.); 4 Diabetes Research Unit, Laval University Medical Research Center, 2705 Boulevard Laurier, Quebec City, QC G1V 4G2, Canada

**Keywords:** gestational diabetes, healthy diet, obesity, type 2 diabetes

## Abstract

Children born from mothers with gestational diabetes mellitus (GDM) are at high-risk of obesity and type 2 diabetes. To date, there is a lack of effective strategies to prevent these complications. The aim of this study was to evaluate the association between diet quality and anthropometric and glycemic profiles of children exposed (GDM+) and unexposed (GDM–) to GDM. A total of 104 GDM+ and 38 GDM– children were included. Two 24-h dietary recall questionnaires were used to assess dietary intakes. The Healthy Eating Index adapted for the Canadian population (HEI-C) was used to assess diet quality. Spearman correlations adjusted for children’s age and sex were computed. Mean age was 6.0 ± 2.5 and 6.8 ± 2.3 years for GDM+ and GDM–, respectively (*p* = 0.03). Total HEI-C score was negatively associated with the android-to-gynoid fat mass ratio (*r* = −0.29, *p* = 0.03) and homeostasis model assessment for insulin resistance (HOMA-IR) index (*r* = −0.22, *p* = 0.04) in GDM+ children only. The prevalence of being overweight or obese during childhood was 4-fold higher among GDM+ children with a HEI-C score ≤70 compared to GDM+ children with a HEI-C score >70. Results of this study show that a healthy diet is associated with a better cardiometabolic health profile in GDM+ children, including a lower risk of being overweight or obese.

## 1. Introduction

Gestational diabetes mellitus (GDM), defined as hyperglycemia with first onset or recognition during pregnancy [1], is associated with health consequences for the child exposed in utero [2,3]. Indeed, in utero exposure to high glucose levels enhances fetal growth and fat deposition, and contributes to the high risk of developing obesity later in life [4]. Accordingly, we have previously shown that children born from mothers with GDM (GDM+) have alterations in body fat proportion and distribution at 6 years of age when compared to children unexposed to GDM (GDM–) during pregnancy [5]. In utero exposure to GDM is also associated with a higher risk developing type 2 diabetes later in life through increased fetal insulin secretion and altered pancreatic function [6,7].

Although efforts have been made to prevent GDM development among pregnant women [8,9], there is currently a lack of effective postnatal strategies to prevent obesity and type 2 diabetes among these high-risk children [10]. Although it is well established that a healthy diet is associated with better cardiometabolic health in children [11,12], little is known regarding the impact of diet quality on anthropometric and glycemic profiles among GDM+ children, a group at high risk. 

Assessment of diet quality can be made using various methods. In 2009, a Canadian version of the American Healthy Eating Index (HEI-C) was developed and validated in the Canadian population aged of 2 years or more [13]. This score provides an overall rating of the adherence to the 2007 Canadian Food Guide (CFG) recommendations and therefore, facilitates the evaluation of diet quality. Accordingly, the aim of this study was to assess the association between diet quality, using the HEI-C score, and anthropometric and glycemic profiles of GDM+ and GDM– children. We found that adopting a healthy diet was associated with more favorable anthropometric and glycemic profiles in GDM+ children. Interestingly, overweight or obesity was four-fold less prevalent in children with a high HEI-C score.

## 2. Materials and Methods 

### 2.1. Study Population

All children were participants of a cohort study that aims to evaluate the impact of GDM on maternal and offspring health and to examine whether a healthy postnatal environment could attenuate the adverse consequences of in utero exposure to GDM. This project has been previously described [14]. Briefly, this study is conducted at the Institute of Nutrition and Functional Foods (INAF), at Laval University (Quebec City, Canada), and recruits women with or without a history of GDM and their children born between 2003 and 2013 and aged between 2 and 14 years in the metropolitan area of Quebec City. Recruitment has been conducted using medical records from the two major hospitals with a neonatal care unit in the area of Quebec City (Hôpital Saint-François d’Assise and Centre Hospitalier de l’Université Laval) and administrative data from the provincial health plan registry (Régie de l’assurance maladie du Québec), as well as email addresses of student and employees of the Laval University Community. Children exposed to GDM in utero were recruited and children exposed to either type 1 or type 2 diabetes during pregnancy were excluded from the project. The majority of children included (92%) were born before 2013, i.e., when GDM was diagnosed with 2003 criteria of Diabetes Canada [15]. Children from the control group had to be born from a mother without a history of GDM, type 1 or type 2 diabetes. Written consent was obtained from all participating mothers and children and ethical approval was obtained from the Laval University Ethics Committee (2011-196-A-4 R-3) and from the Centre Hospitalier Universitaire de Québec Ethics Committee (2015–2031). This cohort study was registered in the Clinical Trials.gov registry (NCT01340924).

### 2.2. Food Assessment

Children came to the INAF research center for a single 1-hour visit with their mother where current dietary intakes were assessed as described previously [16]. Briefly, a 24-h dietary recall questionnaire (24HDR) was conducted with a trained dietician using the multiple-pass method [17]. All items consumed from midnight to midnight the previous day were listed. To enhance accuracy of portion size estimates, three-dimensional food models were used. For children younger than 10 years, the 24HDR was completed with mothers while children were present during the interview and were asked to add information if needed (i.e., for food consumed outside the home). For older children (≥10 years), the 24HDR was conducted directly with the child, with the help of the mother if needed (i.e., for food preparation details). A second 24HDR was conducted over the phone, 7–10 days after the visit at the research center, using the same validated method [17].

The HEI-C score is based on the American Index but is adapted to the Canadian population by using number of servings, according to age and sex, defined in the 2007 version of CFG [13]. The HEI-C score has been validated in the Canadian population aged 2 and over using data from the 2004 Canadian Community Health Survey–Nutrition [13]. Briefly, the HEI-C score is composed of eleven components, which includes eight adequacy components (total vegetables and fruit, whole fruit, dark green and orange vegetables, total grain products, whole grains, milk and alternatives, meat and alternatives, and unsaturated fats) and three moderation components (saturated fats, sodium, and “other food”) [13]. Adequacy components represent food items that should be consumed in high amounts in order to achieve a healthy diet (high intakes are associated with high adequacy scores) whereas moderation components represent foods that should be limited in a healthy diet (low intakes are associated with a high moderation score). The maximal possible score is 100, with adequacy components contributing to 60 points and moderation components to 40 points [13]. Good diet quality is defined as a score >80; a score between 50 and 80 corresponds to diet that requires improvement while a score <50 is associated with poor diet quality [13].

Mean intakes from the two 24HDR were used to calculate the HEI-C total score and its components. Number of servings per day for each food group (vegetables and fruit, grain products, milk and alternatives, meat and alternatives) according to 2007 CFG serving size were calculated [18]. The whole fruit component was obtained by excluding fruit juice from the total fruit group [19]. However, fruit juice was part of the total vegetables and fruit group. The 2007 Canadian classification was used to calculate the whole grains component as well as the dark-green and orange vegetables component, which includes some orange fruits rich in vitamin A such as apricots, peaches, cantaloupes, mango, nectarines, and papayas [20]. The “other food” component was derived from a list of foods and beverages that are mostly fats and sugars, as described in the CFG [18]. Unsaturated fats, saturated fats, and sodium components were extracted using Nutrition Data System for Research software (NDSR version 2011, Nutrition Coordinating Center, University of Minnesota, Minneapolis, MN, USA).

### 2.3. Outcomes

Height was measured to the nearest 0.1 centimeter using a stadiometer, weight was measured to the nearest 0.1 kilogram using a calibrated balance (Tanita BC-418) and body mass index (BMI) was calculated (kg/m^2^). BMI *z* score was calculated using World Health Organization (WHO) Anthroplus software (version 1.0.4, WHO, Geneva, Switzerland). A BMI *z* score >2 for children younger than 5 years and BMI *z* score >1 for children of 5 years or more were used to define overweight or obesity status according to the WHO classification [21,22]. Waist circumference was measured twice, at the umbilical level, to the nearest millimeter and the mean of the two measurements was used in analyses [23]. Body composition was measured by dual-energy X-ray absorptiometry scanner (DEXA, GE Healthcare Lunar; Madison, WI, USA). Fat mass percentage and fat mass distribution (gynoid and android fat mass percentage) of children were analyzed as previously described [5]. The android-to-gynoid fat mass ratio was calculated. Blood samples were collected after a 12-h fast. Plasma glucose was measured enzymatically [24] and plasma insulin was measured by electrochemiluminescence (Roche Diagnostics, Indianapolis, USA). To measure glycated hemoglobin (HbA1c) levels, the Cobas Integra 800 standardized to the National Glycated Hemoglobin Standardization Program (Integra Inc., Roche, Switzerland) was used. The homeostasis model assessment for insulin resistance (HOMA-IR) index was calculated as follows: ((Fasting insulin (pmol/L)/6.945) × fasting glucose (mmol/L))/22.5 [25].

### 2.4. Other Measurements

Socioeconomic data were collected by a self-administered questionnaire completed by the mother. Maternal GDM status and the type of treatment used during pregnancy (i.e., medication (98.3% insulin and 1.7% oral hypoglycemic agents) or diet) were self-reported by the mother. Actual maternal body weight was measured to the nearest 0.1 kg using a calibrated balance (Tanita BC-418), height was measured on a standing position to the nearest millimeter and BMI was calculated.

### 2.5. Statistical Analyses

Descriptive characteristics were calculated as mean ± standard deviation or as percentage (%). Participant’s characteristics and diet quality were compared between the GDM+ and GDM– groups using Student’s *t*-test, chi-squared test (*χ^2^*), and ANOVA. Spearman rank correlations were computed to assess the association between diet quality (HEI-C score and its components) and anthropometric or glycemic profiles of GDM+ and GDM– children. Adjustments for children’s age and sex were performed. Prevalence ratio (PR) using log binominal models were calculated to evaluate the prevalence of overweight or obesity in GDM+ children with a diet quality under the median HEI-C score (i.e., ≤70), compared to GDM+ children with a HEI-C score above the median. The low prevalence of children with a good quality diet (i.e., score >80) did not allow us to compare the prevalence of overweight or obesity according to this cut-off. Furthermore, given the small number of GDM– children with overweight or obesity (*n* = 3), PR for this group was not computed. Finally, the proportion of overweight or obese children according to the median HEI-C score have been compared using a *χ^2^* test. A *p*-value <0.05 and confidence interval (CI) of 95% were used to determine statistical significance. The statistical software SAS studio was used (SAS Institute Inc., Cary, NC, United States).

## 3. Results

### 3.1. Participant’s Characteristics

A total of 104 GDM+ and 38 GDM– children with available nutritional data were included in this study. As shown in Table 1, GDM+ children were younger than GDM– children (6.0 ± 2.5 and 6.8 ± 2.3 years, respectively, *p* = 0.03). Furthermore, GDM+ children presented altered anthropometric and glycemic profiles compared to GDM– children as published previously (Table 1) [5].

There was no difference between mean HEI-C score in GDM+ and GDM– children, although mean intakes of components of the score were different between groups (Table 2). In fact, GDM+ children ate less whole fruits and grain products and more meat and alternatives as well as saturated fats than GDM–children (*p* < 0.05, Table 2).

### 3.2. Diet Quality and Anthropometric and Glycemic Profiles of Children

Among GDM+ children, the total HEI-C score was negatively associated with android-to-gynoid fat mass ratio (*r* = −0.29, *p* = 0.03) and HOMA-IR index (*r* = −0.22, *p* = 0.04) (Table 3 and Table 4), but was not significantly associated with anthropometric or glycemic profiles among GDM– children (Table 3 and Table 4). Components of the HEI-C score were associated differently to anthropometric and glycemic profiles in GDM+ versus GDM– children (Table 3 and Table 4). More specifically, higher intakes of total grain products as well as milk and alternatives were associated with a less central adipose tissue distribution among GDM+ children. Lower intake of foods rich in fats and sugars (higher “other foods” score) was associated with lower HbA1c levels in GDM+ children (*p* < 0.05, Table 4). Among GDM– children, higher intakes of total grains as well as whole grains were associated with lower gynoid fat mass percentage (*p* < 0.05, Table 3). In addition, among GDM– children, higher intakes of whole fruits, dark green or orange vegetables as well as unsaturated fats were associated with a better anthropometric profile (*p* < 0.05, Table 3).

Finally, the prevalence of being overweight or obese during childhood was 4-fold higher among GDM+ children with a low HEI-C score, i.e., ≤70, compared to GDM+ children with a HEI-C score above 70 (PR: 4.01, confidence interval (1.21–13.26). Furthermore, a higher proportion of overweight or obese children had a diet quality below the median HEI-C score of 70 (Table 5).

## 4. Discussion

Results of this study suggest that a healthy diet is associated with better anthropometric and glycemic profiles among GDM+ children. Indeed, the total HEI-C score was negatively associated with the android-to-gynoid fat mass ratio and HOMA-IR index in GDM+ children. Furthermore, the prevalence of being overweight or obese during childhood was 4-fold higher among GDM+ children with a HEI-C score ≤70 compared to GDM+ children with a HEI-C score >70. Finally, higher scores of total grain products as well as milk and alternatives scores were associated with a better anthropometric profile, including a more favorable adipose tissue distribution, i.e., less central adipose tissue distribution, and lower intakes of sugars and fats were associated with lower HbA1c values among GDM+ children. 

We showed that a healthy diet, as assessed with the total HEI-C score, was associated with a more favorable adipose tissue distribution, i.e., with a lower android-to-gynoid fat mass ratio, and a lower insulin resistance state among GDM+ children. In addition, among GDM+ children, a HEI-C score ≤70 was associated with an increased prevalence of overweight or obesity when compared to a HEI-C score >70. To our knowledge, this is the first study assessing the association between diet quality and cardiometabolic health of GDM+ children, which limits comparisons with the current literature. However, this is consistent with a study from the general population. Indeed, among 630 Canadian children at risk of overweight from the Quebec Adiposity and Lifestyle Investigation in Youth (QUALITY) cohort, poor diet quality, defined as a “fast food” eating pattern, was associated with overweight and higher adiposity measurements during childhood [26]. In a similar manner, it has been shown that an energy-dense, high-fat, low-fiber diet was associated with greater adiposity levels or higher odds of excess adiposity in childhood [27,28]. Moreover, in a sample of children from Iran, the total HEI score was negatively associated with insulin resistance state [29]. 

This study also suggests that grain products were associated with a better anthropometric profile in both groups. These results are consistent with results from the National Health and Nutrition Examination Survey III study showing that adolescents in the lowest quartile of waist circumference consumed one more serving of grain products per day, compared to those in the highest quartile of waist circumference [30]. In that previous study, waist circumference was negatively associated with both whole- and refined-grain consumption [30]. Furthermore, prospective data from the Women, Infants and Children (WIC) program showed that each increase of one serving of breads and grains per day was associated with a decrease of 0.16 kg in infant and children bodyweight [31].

We also found that milk and alternatives component was negatively associated with android-to-gynoid fat mass ratio among GDM+ children, suggesting a better fat mass distribution with higher intakes of milk and alternatives. In the current literature, the association between dairy intakes and body composition of children is controversial [32]. Indeed, results from a systematic review and a meta-analysis showed no significant association between dairy intakes and adiposity measures in pre-school- and school-aged children, although a modest protective effect of dairy consumption on adiposity measurements was found in adolescents [33]. Similarly, results from a systematic review of randomized controlled trials concluded that there were no significant associations between dairy consumption and body composition among children and adolescents [34]. The inconsistent association between dairy consumption and body composition could be explained by the fact that results vary according to various factors including the type of dairy products (i.e., low- vs. high fat) and amount consumed, age of children as well as their health status [32]. Further investigation is needed to understand the role of dairy products consumption on adipose tissue distribution among high-risk children such as those exposed in utero to GDM.

Finally, results of this study showed that scores related to whole fruits, dark green or orange vegetables and unsaturated fats were associated with a better anthropometric profile in GDM–, but not in GDM+ children. Indeed, a higher whole fruit score was associated with lower BMI *z* score, waist circumference, fat mass %, android and gynoid fat mass as well as with a lower android-to-gynoid fat mass ratio among GDM– children. The lack of association between the whole fruit score and anthropometric profile among GDM+ children could be explained in part by the fact that GDM+ children consumed significantly less whole fruits than GDM– children, which may have attenuated the potential effect of fruits consumption on children’s health. However, further investigation is needed to better understand this finding. Similarly, unsaturated fats score was associated with lower android, gynoid and total body fat mass percentage as well as lower android-to-gynoid fat mass ratio in GDM– children only. The absence of association in the GDM+ group compared to GDM– group could be explained by the type of food containing unsaturated fats and consumed by these children that could be different between groups. In fact, as shown by Joyce et al. in a cohort study of children from 5 to 12 years, sources of monounsaturated and polyunsaturated fats in children’s diet come from a variety of foods with different nutritional content, such as fish and fried potatoes [35]. Thus, nutritional matrix of these different sources of unsaturated fat could influence differently children’s health. However, this hypothesis needs to be confirmed.

It seems that we observed stronger associations between diet quality and the anthropometric profile than with the glycemic profile. This could be explained by the young age of participants included in our study. In fact, results from a study conducted among offspring of women with preexisting diabetes or GDM showed that only a small proportion of children presented an impaired glucose tolerance (IGT) before 10 years of age (1.2% in children <5 years and 5.4% in children aged 5–9 years), while the prevalence of IGT reached 19.3% in children aged between 10 and 16 years [36]. Thus, further studies should evaluate the association between diet quality and glycemic profile of older GDM+ children in order to better understand this relation.

Although this study highlights the importance of a healthy diet to prevent anthropometric and glycemic alterations in GDM+ and GDM– children, we cannot exclude the possibility that the association between a healthy diet and children’s health could be mediated by other lifestyle habits, like physical activity [37] or maternal characteristics (BMI, education level). In fact, one limitation of this study is that physical activity has not been included in the analyses, given the limited number of participants that had complete data for this variable. Another limitation of the study is the small number of participants in the control group which limits the comparison between groups. Also, given the difficulty to recruit a large number of participants from a specific population such as children exposed in utero to GDM, participants included in this study had a wide range of ages (i.e., from 2 to 14 years) although the majority were within 2 and 9 years of age. However, adjustment for children’s age were made in order to minimize the impact of this limitation. Finally, these results cannot be extended to the general population given that our sample of participants included mostly Caucasians from high-income families. All analyses should be replicated in a larger cohort study in order to confirm associations observed in the present study. 

On the other hand, our study presents several strengths. First, this is to our knowledge the first study to evaluate the association between diet quality and anthropometric and glycemic profiles of GDM+ children, a specific population at high risk of obesity and type 2 diabetes [2,3,5]. Given the lack of effective strategy to prevent these complications in this population, our study addresses the gap observed in the current literature. In addition, while a day-to-day variation in dietary intakes linked to 24HDR is possible, the use of two 24HDR questionnaires to assess children’s diet reduced this daily variability in dietary intakes while minimizing burden in participants. Finally, the diet quality of children was evaluated using a score that has been validated in the Canadian population aged 2 years or more [13]. The use of a score to assess diet quality is a strong aspect of our study because it reflects a real-life context where foods are not consumed in isolation and can, therefore, have a synergistic effect on children’s health.

## 5. Conclusions

In conclusion, our study showed that a healthy diet is associated with lower anthropometric and glycemic alterations in predisposed children born from mothers with GDM. Particularly, the total HEI-C score was associated with lower prevalence of overweight or obesity and a better adipose tissue distribution profile in GDM+ children. Given that GDM+ children are at high risk of obesity and type 2 diabetes later in life and that few children reached a good quality diet, the importance of adopting a healthy diet should be promoted early in their life in order to prevent metabolic alterations in this high-risk population. 

## Figures and Tables

**Table 1:** Participant characteristics.

Characteristics	Mean ± SD or *n* (%)		
	GDM+	GDM−	*p*-Value
	*n* = 104	*n* = 38	
**Demographics**			
Age (years)	6.0 ± 2.5	6.8 ± 2.3	0.03
2–5 (years)	53 (51.0)	12 (31.6)	0.12
6–9 (years)	41 (39.4)	21 (55.3)	
10–14 (years)	10 (9.6)	5 (13.2)	
Sex			
Boys	53 (51)	16 (42)	0.35
Girls	51 (49)	22 (58)	
BMI *z* score	0.25 ± 1.1	0.05 ± 0.8	0.22
Overweight or obese	16 (15.4)	3 (7.9)	0.12
Waist circumference	55.9 ± 7.8	54.9 ± 5.9	0.007 *
Fat mass (%) ^1^	27.0 ± 6.4	24.7 ± 4.0	0.02 *
Android fat mass (%) ^1^	20.3 ± 9.4	16.7 ± 6.0	0.01 *
Gynoid fat mass (%) ^1^	32.1 ± 7.1	29.5 ± 4.8	0.03 *
Android-to-gynoid fat mass ratio ^1^	0.61 ± 0.2	0.56 ± 0.1	0.03 *
Glycemia (mmol/L) ^2^	4.9 ± 0.5	4.8 ± 0.4	0.27 *
Insulinemia (pmol/L) ^2^	65.0 ± 31.9	56.4 ± 19.7	0.02 *
HbA1C (%) ^3^	5.3 ± 0.3	5.2 ± 0.2	0.39 *
HOMA-IR ^4^	2.1 ± 1.3	1.8 ± 0.7	0.03 *
Maternal age (years)	38.1 ± 4.8	36.2 ± 5.4	0.05
Actual maternal BMI (kg/m^2^)	26.8 ± 6.6	23.9 ± 4.1	0.006
GDM treatment during pregnancy ^5^			
Diet	35 (37.6)	-	-
Medication (insulin or oral hypoglycemic agents)	58 (62.4)	-	
Family income (CAD$/year) ^6^			
0–39,000	11 (13.3)	7 (22.6)	0.41
40,000–79,000	24 (28.9)	9 (29.0)	
80,000–99,999	21 (25.3)	4 (12.9)	
>100,000	27 (32.5)	11 (35.5)	
Maternal education level ^7^			
High school or less	17 (18.5)	4 (12.5)	0.71
College	18 (19.6)	6 (18.8)	
University	57 (62.0)	22 (68.8)	
Parity (number of living children)	2.2 ± 0.8	2.2 ± 0.8	0.79

^1^*n* = 56 for children exposed to GDM (GDM+) and *n* = 30 for children unexposed to GDM (GDM–), ^2^ *n* = 94 for GDM+ and *n* = 33 for GDM–, ^3^ *n* = 93 for GDM+ and *n* = 34 for GDM–, ^4^ *n* = 94 for GDM+ and *n* = 32 for GDM–, ^5^ *n* = 93, ^6^ *n* = 83 for GDM+ and *n* = 31 for GDM–, ^7^ *n* = 92 for GDM+ and *n* = 32 for GDM–. BMI: body mass index; GDM: gestational diabetes mellitus; HOMA-IR: homeostasis model assessment for insulin resistance; HbA1C: glycated hemoglobin. * ANOVA adjusted for children’s age and sex.

**Table 2:** Diet quality during childhood.

	Mean ± SD or *n* (%)		
	GDM+	GDM−	*p*-Value
	*n* = 104	*n* = 38	
HEI-C score			
Total score	68.4 ± 11.7	70.9 ± 11.1	0.25
Poor diet (score <50)	7 (6.7)	1 (2.6)	0.61
Diet that required improvement (≥50 score ≤80)	76 (73.1)	28 (73.7)	
Good quality diet (score >80)	21 (20.2)	9 (23.7)	
Mean intake of each component ^‡^			
Total vegetables and fruit (serving/day)	4.7 ± 2.5	5.4 ± 2.6	0.06
Whole fruit (serving/day)	1.7 ± 1.2	2.2 ± 1.6	0.01
Dark green and orange vegetables (serving/day)	0.9 ± 0.8	0.8 ± 0.8	0.40
Total grain products (serving/day)	4.3 ± 2.0	5.0 ± 1.7	0.04
Whole grains (serving/day)	1.2 ± 1.2	1.5 ± 1.5	0.24
Milk and alternatives (serving/day)	2.3 ± 1.2	2.4 ± 1.1	0.41
Meat and alternatives (serving/day)	1.7 ± 0.9	1.4 ± 0.7	0.03
Unsaturated fats (g/day)	31.4 ± 11.6	32.2 ± 11.7	0.68
Saturated fats (g/day)	12.5 ± 2.9	11.2 ± 2.8	0.03
Sodium (mg/day)	2398 ± 726	2436 ± 816	0.76
Other food (% of energy intake/day)	21.8 ± 11.5	21.5 ± 9.6	0.91

^‡^ Adjustment for children’s age and sex. HEI-C: Canadian version of the American Healthy Eating Index.

**Table 3:** Association between diet quality and anthropometric profile of GDM+ and GDM– children.

GDM+ Children							GDM– Children					
	BMI *Z* Score	Waist Circumference ^‡^	Fat Mass % ^‡,a^	Android Fat Mass (%) ^‡,a^	Gynoid Fat Mass (%) ^‡,a^	Android-to-Gynoid Fat Mass Ratio ^‡,a^	BMI *z* Score	Waist Circumference ^‡,b^	Fat Mass % ^‡,b^	Android Fat Mass (%) ^‡,b^	Gynoid Fat Mass (%) ^‡,b^	Android-to-Gynoid Fat Mass Ratio ^‡,b^
HEI-C score	0.01	−0.06	−0.07	−0.23	−0.07	−0.29 *	−0.25	0.02	−0.25	−0.21	−0.24	−0.20
Adequacy scores												
Total vegetables and fruit	0.17	0.08	0.16	0.07	0.17	−0.02	−0.16	−0.15	0.26	0.24	0.27	0.18
Whole fruit	0.06	−0.009	0.07	0.08	0.12	−0.02	−0.50 **	−0.44 **	−0.39 *	−0.46 *	−0.38 *	−0.41 *
Dark green and orange vegetables	0.02	−0.02	−0.10	−0.16	−0.10	−0.23	−0.43 **	−0.19	−0.11	−0.12	−0.14	−0.20
Total grain products	−0.21 *	−0.14	−0.10	−0.35 **	−0.21	−0.36 **	−0.12	−0.11	−0.35	−0.30	−0.43 *	−0.19
Whole grains	0.06	0.03	−0.15	−0.22	−0.14	−0.26	−0.17	0.08	−0.31	−0.21	−0.41 *	−0.07
Milk and alternatives	−0.13	0.11	−0.15	−0.26	−0.14	−0.28 *	0.11	0.13	−0.13	−0.24	−0.11	−0.23
Meat and alternatives	0.03	0.009	−0.001	−0.08	−0.09	−0.10	0.12	0.12	0.11	0.08	−0.07	0.15
Unsaturated fats	0.05	0.20 *	0.16	0.09	0.16	0.10	0.002	−0.06	−0.43 *	−0.44 *	−0.44 *	−0.40 *
Moderation scores												
Saturated fats	−0.02	−0.10	0.22	0.17	0.20	0.14	−0.02	0.23	0.18	0.27	0.16	0.31
Sodium	−0.08	−0.24 *	−0.26	−0.18	−0.15	−0.21	0.07	0.11	0.17	0.20	0.11	0.20
“Other food”	0.01	−0.05	−0.14	−0.24	−0.15	−0.20	−0.03	−0.01	−0.13	−0.20	−0.10	−0.24

Values are Spearman’s rank coefficient of correlation. * *p* < 0.05, ** *p* < 0.01. ^‡^ Adjustment for children’s age and sex. ^a^ *n* = 56, ^b^ *n* = 30.

**Table 4:** Association between diet quality and glycemic profile of GDM+ and GDM– children.

	GDM+ Children					GDM– Children		
	Glycemia ^‡,a^	Insulinemia ^‡,a^	HOMA-IR ^‡,a^	HbA_1c_^‡,b^	Glycemia ^‡,c^	Insulinemia ^‡,c^	HOMA-IR ^‡,d^	HbA_1c_^‡,e^
HEI-C score	−0.11	−0.22 *	−0.22 *	−0.18	−0.07	0.007	0.03	0.22
Adequacy scores								
Total vegetables and fruit	0.09	−0.03	−0.02	−0.06	0.19	0.24	0.29	0.15
Whole fruit	−0.06	−0.11	−0.10	0.05	−0.03	0.11	0.12	0.03
Dark green and orange vegetables	0.08	0.01	0.03	−0.13	0.13	<0.001	0.13	0.28
Total grain products	−0.07	−0.02	−0.03	−0.18	−0.009	-0.18	−0.08	0.11
Whole grains	0.06	−0.02	−0.02	−0.10	−0.05	0.33	0.27	0.29
Milk and alternatives	0.03	0.06	0.04	−0.10	−0.05	−0.06	−0.07	0.18
Meat and alternatives	−0.04	−0.007	0.003	−0.03	−0.44 *	−0.30	−0.32	−0.11
Unsaturated fats	−0.08	−0.11	−0.12	−0.12	0.02	0.07	0.09	0.08
Moderation scores								
Saturated fats	0.15	−0.03	−0.003	0.02	0.13	0.08	0.10	0.13
Sodium	−0.15	−0.20	−0.19	0.16	−0.09	0.02	−0.09	−0.16
“Other food”	−0.10	−0.14	−0.15	−0.22 *	−0.04	0.13	0.13	−0.16

Values are Spearman’s rank coefficient of correlation. * *p* < 0.05, ** *p* < 0.01. ^‡^ Adjustment for children’s age and sex. ^a^ *n* = 94, ^b^ *n* = 93, ^c^ *n* = 33, ^d^ *n* =32, ^e^ *n* = 34.

**Table 5:** Prevalence of being overweight or obese during childhood among GDM+ according to diet quality.

	Normal Weight	Overweight or Obese	*p-*Value
HEI-C score >70	47 (39.4)	3 (2.9)	**0.01**
HEI-C score ≤70	41 (45.2)	13 (12.5)	

Results are presented as follows: *n* (%).
